# Comparative genomic analysis of *Thermus* provides insights into the evolutionary history of an incomplete denitrification pathway

**DOI:** 10.1002/mlf2.12009

**Published:** 2022-04-29

**Authors:** Jian‐Yu Jiao, Zheng‐Han Lian, Meng‐Meng Li, Nimaichand Salam, En‐Min Zhou, Lan Liu, Hong Ming, Guoxing Nie, Wensheng Shu, Guoping Zhao, Brian P. Hedlund, Wen‐Jun Li

**Affiliations:** ^1^ State Key Laboratory of Biocontrol, Guangdong Provincial Key Laboratory of Plant Resources and Southern Marine Science and Engineering Guangdong Laboratory (Zhuhai), School of Life Sciences Sun Yat‐Sen University Guangzhou China; ^2^ International Joint Research Center for Karstology, School of Earth Sciences Yunnan University Kunming China; ^3^ Synthetic Biology Engineering Laboratory of Henan Province, College of Life Sciences and Technology Xinxiang Medical University Xinxiang China; ^4^ College of Fisheries Henan Normal University Xinxiang China; ^5^ Institute of Ecological Science, School of Life Science South China Normal University Guangzhou China; ^6^ Institute of Synthetic Biology, Shenzhen Institutes of Advanced Technology Chinese Academy of Sciences Shenzhen China; ^7^ School of Life Sciences University of Nevada Las Vegas Nevada USA; ^8^ Nevada Institute of Personalized Medicine University of Nevada Las Vegas Nevada USA; ^9^ State Key Laboratory of Desert and Oasis Ecology Xinjiang Institute of Ecology and Geography, Chinese Academy of Sciences Urumqi China.

**Keywords:** comparative genomics, denitrification, evolutionary history, *Thermus*

## Abstract

Biological denitrification is a crucial process in the nitrogen biogeochemical cycle, and *Thermus* has been reported to be a significant heterotrophic denitrifier in terrestrial geothermal environments. However, neither the denitrification potential nor the evolutionary history of denitrification genes in the genus *Thermus* or phylum *Deinococcota* is well understood. Here, we performed a comparative analysis of 23 *Thermus* genomes and identified denitrification genes in 15 *Thermus* strains. We confirmed that *Thermus* harbors an incomplete denitrification pathway as none of the strains contain the *nosZ* gene. Ancestral character state reconstructions and phylogenetic analyses showed that *narG*, *nirS*, and *norB* genes were acquired by the last common ancestor of *Thermales* and were inherited vertically. In contrast, *nirK* of *Thermales* was acquired via two distinct horizontal gene transfers from *Proteobacteria* to the genus *Caldithermus* and from an unknown donor to the common ancestor of all known *Thermus* species except *Thermus filiformis*. This study expands our understanding of the genomic potential for incomplete denitrification in *Thermus*, revealing a largely vertical evolutionary history of the denitrification pathway in the *Thermaceae*, and supporting the important role for *Thermus* as an important heterotrophic denitrifier in geothermal environments.

## Impact statement

For decades, *Thermus* was always considered to be aerobic. However, recent studies have suggested that the denitrification abilities of *Thermus* species may be widely underestimated. In the present study, we used comparative genomic analysis to investigate the evolutionary history of the denitrification pathway in *Thermus* and other members of the phylum *Deinococcota*. We revealed incomplete denitrification pathways to be common in *Thermus* and showed they are inherited mostly vertically, which further supports the importance of *Thermus* as a significant denitrifier in hydrothermal environments.

## INTRODUCTION

*Thermus* species are members of the family *Thermaceae* and are generally Gram‐strain‐negative, high‐G + C‐content, nonmotile, rod‐shaped, and obligately aerobic or facultatively anaerobic. The genus *Thermus* belongs to the phylum *Deinococcota*, along with the genera *Calidithermus*, *Marinithermus*, *Meiothermus*, *Oceanithermus*, *Rhabdothermus*, *Vulcanithermus*, *Deinococcus*, *Deinobacterium*, and *Truepera*. To date, the genus *Thermus* comprises 19 validly published species names under the International Code of Nomenclature of Prokaryotes (ICNP)^1^, and members of this genus are archetypal thermophilic bacteria that are readily isolated from terrestrial geothermal environments, especially circumneutral pH or alkaline hot springs^2^. *Thermus* species are well known for their biotechnological applications, such as the production of thermostable enzymes (e.g., *Taq* DNA polymerase)^3^. Several species of *Thermus* were reported to have incomplete denitrification pathways with corresponding experimental evidence for the production of nitrite or nitrous oxide^4^, ^5^.

Denitrification is a crucial component of the nitrogen cycle in geothermal systems. Complete biological denitrification is a respiratory process that reduces nitrogenous oxides to dinitrogen and is normally catalyzed by nitrate reductase (NarGHI or NapAB), nitrite reductase (NirK or NirS), nitric oxide reductase (NorBC), and nitrous oxide reductase (NosZ) under oxygen‐limiting or anaerobic conditions^2^. Previous studies have shown that denitrification is highly active in hot springs and that members of the genus *Thermus* play a significant role as heterotrophic denitrifiers in hot spring environments^4^, ^6^. However, the distribution of denitrification genes within *Thermus* and the evolutionary history of the *Thermus* denitrification pathway have not been investigated.

Currently, only a few strains of the genus *Thermus* have been reported to be incomplete denitrifiers, and there is no consensus in the literature on the nature of denitrification in the genus *Thermus*. Furthermore, considering the high plasticity of *Thermus* genomes^7^, natural competence to transform DNA^8^, and abundance of insertion sequence (IS) elements and prophages on *Thermus* genomes, including the plasmid‐borne nitrate conjugative element in *Thermus thermophilus* HB8 and NAR1^5^, ^9^, ^10^, ^11^, we hypothesize that denitrification genes might be obtained through various horizontal gene transfer (HGT) events. If so, the distribution and evolutionary origins of denitrification genes in various *Thermus* species might be highly variable and responsive to selective forces in individual geothermal systems, including the supply of oxidized nitrogen species.

To test our hypothesis, we performed a comparative genome analysis of *Thermus* species and analyzed three aspects of the evolutionary history of the *Thermus* denitrification pathway viz. (1) the presence of genes involved in denitrification pathway in a representative of all *Thermus* species; (2) phylogenetic analysis of major denitrification proteins; and (3) investigation of gene gain and loss events in *Thermus* genomes.

## RESULTS AND DISCUSSION

### Phylogenetic analysis shows monophyly of the genus *Thermus*

**Figure 1:**
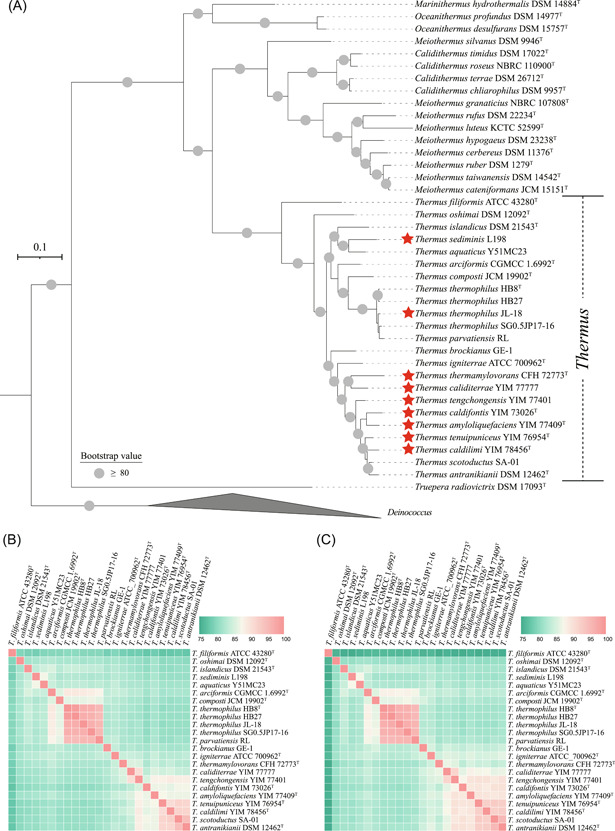
Phylogenetic inference of *Thermus* strains**.** (A) Phylogenetic placement of *Thermus* strains. Multiple sequence alignments of 120 bacterial marker genes were generated by GTDB‐Tk and IQ‐Tree was used to construct the phylogenomic tree. Red stars illustrate that the strains were isolated or the genomes were sequenced previously. (B) Average nucleotide identity (ANI) values shared among the *Thermus* species. (C) Average amino acid identity (AAI) values shared among the *Thermus* species. The ANI and AAI values are presented in Tables S1–S2, respectively.

The genus *Thermus* presently harbors 19 species, most of which can be isolated from terrestrial geothermal environments. A phylogenetic dendrogram indicating the phylogenetic relationships within the phylum *Deinococcota* was generated based on a concatenated alignment of conserved positions based on 120 bacterial marker genes, as implemented by Genome Taxonomy Database‐Toolkit (GTDB‐Tk)^12^. The phylogenomic tree showed that the genus *Thermus* is monophyletic within the family *Thermaceae* with high bootstrap support (Figure 1A). Both average nucleotide identity (ANI) and average amino acid identity (AAI) values showed large genomic divergences among the *Thermus* genomes (Figure 1B,C and Tables S1–S2). However, among the 23 *Thermus* strains, *Thermus thermophilus* and *Thermus parvatiensis* formed a monophyletic clade, and both ANI and AAI values between these two species were higher than 95%, indicating they belong to the same species^13^, ^14^. Given the priority of the species *T. thermophilus*, we suggest that *T. parvatiensis* is a later heterotypic synonym of *T. thermophilus*. The independence of all other *Thermus* species was confirmed using a 95% ANI species boundary^15^. The evolutionary distances between *Thermus*  *filiformis* ATCC 43280^T^ and the other *Thermus* strains were high, although *T. filiformis* ATCC 43280^T^ was monophyletic with other members of the genus *Thermus* with high bootstrap support (Figure 1 and Tables S1–S2).

### Comparative genome analysis of *Thermus* shows similar genomic features

The properties and statistics of the finished and draft *Thermus* genomes are summarized in Table 1. Twenty‐three *Thermus* genomes with 20 different species names ranged in size from 2.02 to 2.56 Mbp, with G + C content between 64.81% and 69.50%. The number of protein‐coding genes ranged from 2224 to 2749, while the number of predicted transfer RNA (tRNAs), encoding almost all 20 amino acids, ranged from 43 to 53. The estimated completeness of all the genomes based on conserved marker genes was more than 99% (except *Thermus amyloliquefaciens* YIM 77409^T^ at 97.81% and *T. parvatiensis* RL at 95.76%). The genome sizes of *Thermus* species were smaller than those of most members within the phylum *Deinococcota*, and the coding density of *Thermus* was higher compared with most other species (Table S3). The smaller genome sizes of *Thermus* reflect a well‐documented pattern of genomic reduction among thermophiles^16^. Mobile genetic elements such as IS elements and genomic islands (GI) were also detected. Each *Thermus* genome contains a large number of IS elements (Table S4), and almost all the genomes (22/23) contain a GI (Table S5). However, no denitrification genes were detected close to any of the mobile genetic elements, suggesting that the denitrification genes might not be transferred through these two mobilomes. The detection of CRISPR‐Cas system indicated their bacterial immune system for protection against alien DNA.

**Table 1:** General features of the *Thermus* genomes.

Strain	Completeness (%)	Contamination (%)	Genome size (bp)	Scaffolds	*N*_50_ (scaffolds)	GC (%)	Coding density (%)	Predicted genes	Unique genes	tRNAs	rRNA copies	CRISPR
*Thermus amyloliquefaciens* YIM 77409^T^	97.81	0	21,60,855	6	20,55,291	67.44	92.61	2315	129	48	2	5
*Thermus antranikianii* DSM 12462^T^	100.00	0	21,65,150	33	2,18,058	64.81	94.46	2265	41	47	1	0
*Thermus aquaticus* Y51MC23	100.00	0	23,38,641	5	21,58,963	68.04	93.15	2501	145	53	2	10
*Thermus arciformis* CGMCC 1.6992^T^	100.00	0	24,42,297	88	58,107	68.79	93.70	2643	105	47	2	12
*Thermus brockianus* GE‐1	99.36	0	23,88,273	3	20,35,182	66.90	93.27	2530	132	47	2	9
*Thermus caldifontis* YIM 73026^T^	100.00	0	21,63,786	41	1,93,267	64.85	94.27	2275	52	46	1	4
*Thermus caldilimi* YIM 78456^T^	99.58	0	24,47,659	1	24,47,659	65.14	93.87	2639	155	47	2	0
*Thermus caliditerrae* YIM 77777	100.00	0.42	22,18,114	4	20,46,548	67.22	93.83	2285	76	50	2	5
*Thermus composti* JCM 19902^T^	100.00	0	20,80,570	64	82,383	67.70	94.64	2247	54	45	1	3
*Thermus filiformis* ATCC 43280^T^	99.15	0.42	23,86,081	40	5,51,922	69.01	92.76	2410	188	47	2	15
*Thermus igniterrae* ATCC 700962^T^	99.58	0.42	22,25,983	74	62,194	68.80	94.94	2331	52	43	2	10
*Thermus islandicus* DSM 21543^T^	99.15	0	22,63,010	67	2,41,998	68.35	93.75	2412	145	47	2	2
*Thermus oshimai* DSM 12092^T^	100.00	0	22,60,954	24	2,00,705	68.74	94.80	2355	93	48	1	15
*Thermus parvatiensis* RL	95.76	0	20,16,098	2	18,72,821	68.53	90.78	2463	189	47	2	2
*Thermus scotoductus* SA‐01	100.00	0	23,55,186	2	23,46,803	64.89	94.25	2458	80	48	2	5
*Thermus sediminis* L198	99.15	0	21,60,271	4	20,28,157	68.21	92.46	2287	149	48	2	5
*Thermus tengchongensis* YIM 77401	100.00	2.54	25,62,314	5	22,24,342	66.40	92.57	2749	179	47	2	5
*Thermus tenuipuniceus* YIM 76954^T^	100.00	0	22,61,036	66	51,325	65.54	94.02	2375	76	47	1	5
*Thermus thermamylovorans* CFH 72773^T^	99.58	0	22,50,808	54	1,71,757	69.50	94.05	2336	89	45	1	11
*Thermus thermophilus* HB27	99.58	0	21,27,482	2	18,94,877	69.41	93.86	2224	43	48	2	10
*T. thermophilus* HB8^T^	99.58	0	21,16,056	3	18,49,742	69.50	93.80	2224	38	48	2	11
*T. thermophilus* JL‐18	100.00	0	23,11,212	3	19,02,595	68.98	94.11	2459	51	49	2	6
*T. thermophilus* SG0.5JP17‐16	100.00	0.42	23,03,227	2	18,63,201	68.62	93.81	2451	98	48	2	8

**Figure 2:**
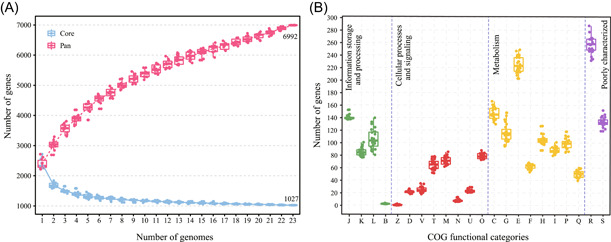
Pan‐ and core‐genome evolution and functional annotations of *Thermus* genomes. (A) Pan‐ and core‐genome evolution of *Thermus*. (B) Categorization of the function of each protein‐coding gene was based on COG categories. J: Translation, ribosomal structure, and biogenesis; K: Transcription; L: Replication, recombination, and repair; B: Chromatin structure and dynamics; Z: Cytoskeleton; D: Cell‐cycle control, cell division, chromosome partitioning; V: Defense mechanisms; T: Signal transduction mechanisms; M: Cell wall/membrane/envelope biogenesis; N: Cell motility; U: Intracellular trafficking, secretion, and vesicular transport; O: Posttranslational modification, protein turnover, chaperones; C: Energy production and conversion; G: Carbohydrate transport and metabolism; E: Amino acid transport and metabolism; F: Nucleotide transport and metabolism; H: Coenzyme transport and metabolism; I: Lipid transport and metabolism; P: Inorganic ion transport and metabolism; Q: Secondary metabolites biosynthesis, transport, and catabolism; R: General function prediction only; S: Function unknown.

The genetic variability among *Thermus* genomes can be further determined from the distribution of the conserved (core) and species‐specific (unique) genes. The pan‐genome of the *Thermus* genomes comprised 6992 gene clusters including 1027 homologous gene clusters in the core genome (Figure 2A). Comparative genome analysis revealed that each of the *Thermus* species contains a small number (38–189) of unique genes in their genomes (Table 1).

The properties and statistics of genes annotated into the cluster of orthologous groups (COGs) functional categories are shown in Figure 2B and Table S6. The most abundant COGs in the *Thermus* genomes are assigned to general function prediction only (COG category R), followed closely by CDSs dedicated to amino acid transport and metabolism (COG category E). These two categories are in higher proportions compared with other COG categories in all *Thermus* genomes. Notably, for an overall comparison among the genomes of *Thermus* strains, all the genomes contain a similar gene number in each COG category, and almost half of the gene clusters (1027) are conserved in all genomes, which suggests that *Thermus* genomes are more stable than generally considered.

### *Thermus* incomplete denitrification pathways evolve through a combination of horizontal and vertical processes

**Figure 3:**
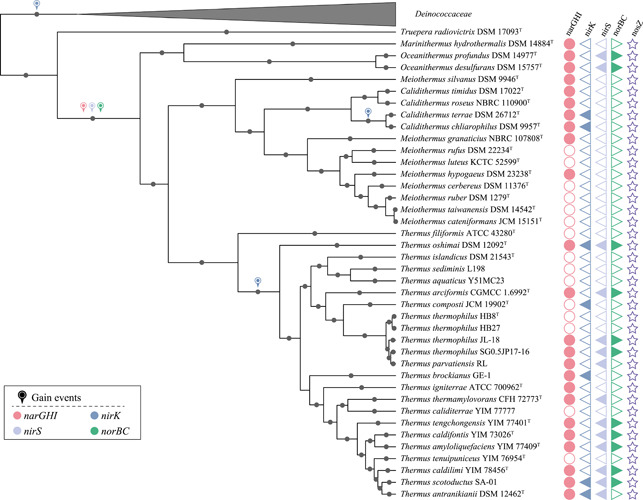
Gain events of denitrification genes in the phylum *Deinococcota*. The Bayesian tree was determined by MrBayes^19^, and gene gain events were calculated by using COUNT software^20^. The black circles represent nodes with posterior possibilities higher than 0.9. The presence or absence of genes related to denitrification genes in the phylum *Deinococcota* is shown on the right of the Bayesian tree. The gain of denitrification genes in *Deinococcota* is marked.

**Figure 4:**
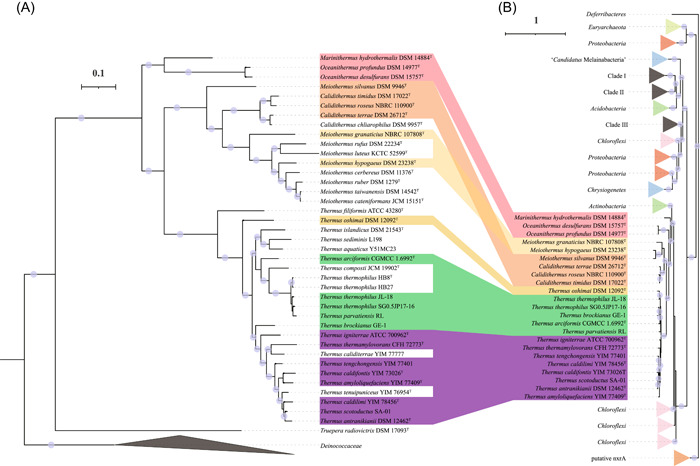
Consistency between the phylogenomic tree of *Deinococcota* and the phylogenetic tree of NarG. (A) The phylogenomic tree of *Deinococcota* was constructed as in Figure 1A. (B) The NarG sequences listed in Table S7 were aligned using MUSCLE with 100 iterations. The NarG tree was constructed using IQ‐Tree with the parameters (‐alrt 1000 ‐bb 1000 ‐nt AUTO). The best‐fit model (LG + R9) was determined by ModelFinder.

Nitrogen oxides can act as the terminal electron acceptors for respiration in the absence of oxygen. Previous studies have reported that the members of *Thermus*, such as *Thermus scotoductus* SE‐1^T^, *Thermus tenuipuniceus* YIM 76954^T^, and a variety of *T. thermophilus* and *Thermus oshimai* strains can grow anaerobically with nitrate as electron acceptors^17^, ^18^. In total, 15 of the examined genomes contained annotated denitrification genes (Figure 3), and the genes coding for denitrification enzymes were located close to genes for cytochrome c oxidase (Figures S1–S2), supporting their potential roles in respiration. Furthermore, the synteny *narGHI*, *nirS*, and *norBC* are conserved, suggesting that these genes evolve as an evolutionary unit (Figures S1–S2). Fourteen of the *Thermus* genomes contain the genes coding for nitrate reductase. Additionally, *narGHI* was prevalent in other genera of *Thermaceae* but was absent from the genera *Truepera* and *Deinococcus*, which belong to the related families *Treuperaceae* and *Deinococcaceae*. Furthermore, phylogenetic analysis of NarG showed that the *Thermales* nitrate reductases were clustered in a single clade (Figure 4), indicating the *narG* genes, overall, were inherited vertically.

Nitrite reduction (NO_2_^−^ to NO) is often a rate‐limiting process in denitrification^21^. Although 14 genomes contain nitrite reductases, the enzymes for nitrite reduction are different in different *Thermus* species. *Thermus* species can catalyze nitrite reduction by two types of nitrite reductases, NirK or NirS. Five *Thermus* strains were shown to contain *nirK*, which encodes the Cu‐containing nitrite reductase (Figure 3), whereas *nirS*, encoding the isofunctional tetraheme cytochrome *cd*1‐containing nitrite reductase, was present in 12 strains. This finding indicates that *nirS*‐type nitrite reductases are more prevalent in *Thermus* species. A previous study reported the distinct roles of *nirK* and *nirS* in the genus *Thermus*: *nirS* was expressed higher in comparison to *nirK* under oxic and stable conditions. In contrast, *nirK* was expressed over a wider range of nitrite concentrations^21^. Three *Thermus* genomes, *T. oshimai* DSM 12092^T^, *T. scotoductus* SA‐01, and *Thermus antranikianii* DSM 12462^T^, harbor two types of nitrite reductases, suggesting that they might be able to utilize nitrite more effectively over a wide concentration range and could use nitrite as an electron acceptor under oxic conditions^21^.

**Figure 5:**
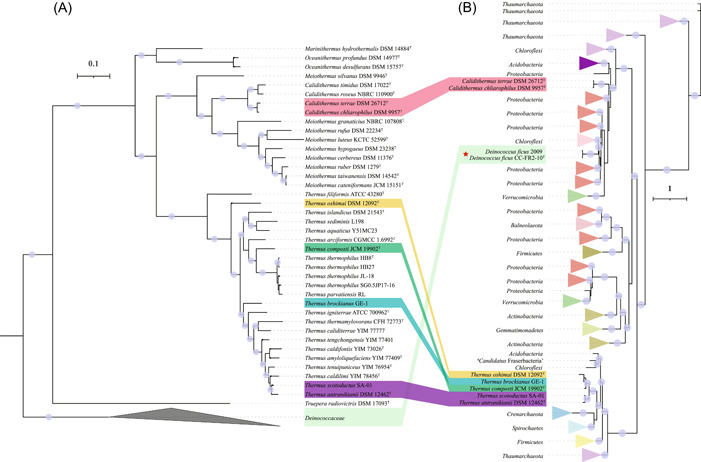
Discrepancy between the phylogenomic tree of *Deinococcota* and the phylogenetic tree of NirK. (A) The phylogenomic tree of *Deinococcota* was constructed as in Figure 1A. (B) The NirK protein sequences listed in Table S8 were aligned using MUSCLE with 100 iterations. The NirK tree was constructed using IQ‐Tree with the parameters (‐alrt 1000 ‐bb 1000 ‐nt AUTO). The best‐fit model (WAG + F + R10) was determined by ModelFinder. Red star illustrates the strains from the family Deinococcaceae.

**Figure 6:**
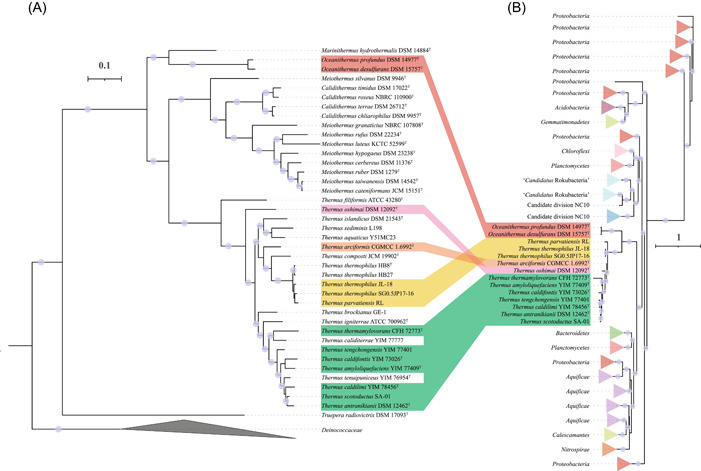
Consistency between the phylogenomic tree of *Deinococcota* and the phylogenetic tree of NirS. (A) The phylogenomic tree of *Deinococcota* was constructed as in Figure 1A. (B) The NirS protein sequences listed in Table S9 were aligned using MUSCLE with 100 iterations. The NirS tree was constructed using IQ‐Tree with the parameters (‐alrt 1000 ‐bb 1000 ‐nt AUTO). The best‐fit model (LG + F + R10) was determined by ModelFinder.

The phylogeny of the NirK from *Thermales* (Figure 5) revealed three major clades, representing the three genera *Thermus*, *Calidithermus*, and *Deinococcus*, which suggests a more complex evolutionary history compared with other denitrification genes. The NirK of the genera *Calidithermus* and *Deinococcus* were clearly within a clade found in *Proteobacteria*, indicating potential HGT events from *Proteobacteria* to these two genera. However, the five *Thermus* NirK proteins were monophyletic, and their sister lineages contained multiple phyla (e.g., *Spirochaetes*, *Firmicutes*, *Acidobacteria*, *Crenarchaeota*, etc.), suggesting a different evolutionary origin. In contrast, NirS was only present in the order *Thermales* and formed a monophyletic clade (Figure 6). Furthermore, the ancestral character state reconstruction of NirS (Figure 3) showed that the last common ancestor of *Thermales* likely encoded NirS for nitrite reduction. Our results suggest that the *Thermales* acquired *nirS* before their divergence, and that *nirS* was subsequently inherited vertically with multiple gene losses; in contrast, *nirK* was acquired from different donors.

**Figure 7:**
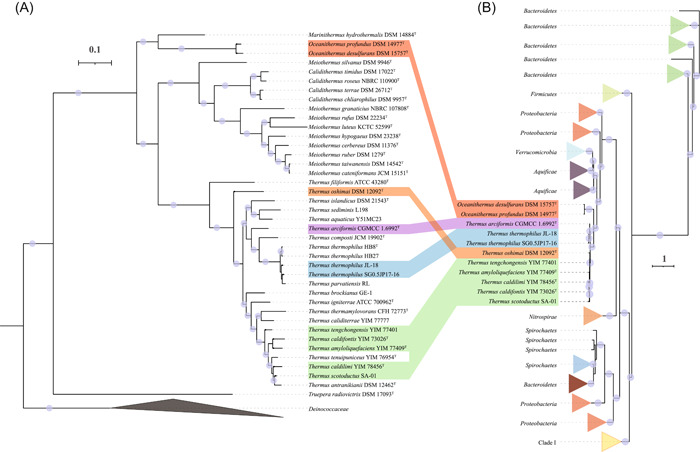
Consistency between the phylogenomic tree of *Deinococcota* and the phylogenetic tree of NorB. (A) The phylogenomic tree of *Deinococcota* was constructed as in Figure 1A. (B) The NorB protein sequences listed in Table S10 were aligned using MUSCLE with 100 iterations. The NorB tree was constructed using IQ‐Tree with the parameters (‐alrt 1000 ‐bb 1000 ‐nt AUTO). The best‐fit model (LG + R10) was determined by ModelFinder.

The genes *norBC*, coding for the nitric oxide reductase, were also detected in the order *Thermales*. In particular, nine *Thermus* genomes and two *Oceanithermus* genomes contain *norBC* (Figure 3). However, no *norBC* was detected in the families *Deinococcaceae* and *Trueperaceae* (Figure 3). The phylogenetic analysis showed that the NorB of *Thermales* formed a single clade (Figure 7), which indicates that *norBC* was present in the common ancestor of the order and was inherited vertically but with multiple gene loss events.

No *nosZ* gene coding for nitrous‐oxide reductase was detected in the *Thermus* genomes (Figure 3), which is consistent with the incomplete denitrification phenotype commonly reported in *Thermus* strains^4^, ^5^. However, one *nosZ* gene was detected in the genome of the type strain of *Deinococcus ficus* CC‐FR2‐10^T^. The NosZ phylogenetic tree showed that it might have been horizontally transferred from *Firmicutes* (Figure S3). The nitrous‐oxide reductase is the most sensitive enzyme in denitrification to oxygen^22^, ^23^, and the absence of *nosZ* in *Thermus* might reflect the niche of *Thermus* in relatively oxidized habitats within thermal environments. All known *Thermus* species are aerobic and tolerant of atmospheric concentrations of oxygen, despite the low solubility of oxygen at high temperatures. Furthermore, most geothermal springs are sourced with ammonium as the dominant source of dissolved organic nitrogen^24^, and aerobic chemolithotrophic ammonia oxidation is thought to be the main process delivering oxidized nitrogen as a source for denitrifiers^6^; the frequent coupling of nitrification and denitrification might select for oxygen tolerance and therefore select against nitrous‐oxide reductase, consistent with high nitrous oxide fluxes measured in some geothermal systems^4^.

In total, the phylogenetic analyses of *Thermus* denitrification proteins were not always consistent with phylogenomic trees (Figures 1A and 4, 5, 6, 7), suggesting some elements of nonvertical evolution. In addition to the complex evolution of NirK, the denitrification enzymes (NarG, NirS, and NorB) from *T. oshimai* DSM 12092^T^ consistently branched in the middle of the clade of the *Thermus* enzymes. This position contrasts with the phylogenomic analyses in which *T. oshimai* DSM 12092^T^ is deep branching within the genus *Thermus* (Figure 1A). The reason may be due to a recent HGT event leading to the gain of the denitrification gene cluster in *T. oshimai* DSM 12092^T^ from a *Thermus* donor, as suggested by a previous study^25^, or due to the low phylogenetic resolution of single genes at the highest and lowest taxonomic ranks^26^.

The emerging view of the evolution of denitrification genes in *Thermales* is one in which the last common ancestor of the order gained an incomplete denitrification gene cluster containing *narGHI*, *nirS*, and *norBC*, which was generally vertically inherited, but with multiple gene losses, particularly for *nirS* and *norBC*. A completely separate evolution is inferred for NirK. Although the genera *Thermus*, *Calidithermus*, and *Deinococcus* (*Deinococcales*) contain *nirK*, phylogenetic analysis of NirK showed that these proteins are not closely related among the genera and were thus gained through independent HGT events. Further back, we infer that the last common ancestor of the *Deinococcota* did not contain genes for complete denitrification, and the *Thermales* acquired these genes early in their evolutionary history, allowing them to adapt to low‐oxygen or anoxic conditions where nitrification or other processes provide oxidized nitrogen species to support denitrification.

In summary, we first analyzed the distribution and evolution of denitrification genes in *Deinococcota* genomes and provided insights into the evolutionary history of the *Thermus* denitrification pathway. Similar to other studies, our results show that the *Thermus* genomes contain an incomplete denitrification pathway; more than half of the investigated *Thermus* genomes contain one or more denitrification genes, and none of them contain the *nosZ*. The phylogenetic analysis revealed distinct evolutionary histories of *narGHI*, *nirS*, and *norBC* versus *nirK*, and demonstrated that the last common ancestor of the *Thermales* acquired these genes early in their evolutionary history, and these genes were largely inherited vertically. Our results support the incomplete denitrification pathway in *Thermus* and suggest that this pathway allowed it to adapt to the low‐oxygen or anoxic conditions that are common in geothermal environments, which also expand our knowledge of the evolution of the denitrification pathway in *Thermus* and support the importance of *Thermus* as a significant heterotrophic denitrifier in thermal environments.

## MATERIALS AND METHODS

### Genome sequencing, assembly, and annotation

The genomes of *T. tenuipuniceus* YIM 76954^T^, *T. sediminis* L198, *T. caliditerrae* YIM 77777, *T. tengchongensis* YIM 77401, *Thermus caldifontis* YIM 73026^T^, *T. amyloliquefaciens* YIM 77409^T^, *T. thermophilus* JL‐18, *T. caldilimi* YIM 78456^T^, and *T. thermamylovorans* CFH 72773^T^ were sequenced and assembled as reported earlier^2^, ^17^, ^27^, ^28^, ^29^, ^30^, ^31^, ^32^. Other reference genomes of *Thermus* species were downloaded from the NCBI database (Table S3). The habitat of these strains was present in Table S3, and the completeness and contamination of each genome were calculated using CheckM^33^. Putative protein‐coding sequences (CDSs) of each genome were predicted using Prodigal^34^ with the “‐p single” parameter, and the CDSs were annotated against eggNOG, KEGG, and NCBI‐nr databases using DIAMOND^35^ by applying *E*‐values < 1e−5. The predicted CDSs were also uploaded to KEGG Automatic Annotation Server (KAAS)^36^ with “bidirectional best hit” and “for prokaryotes” parameters. The tRNAs and rRNAs were identified by tRNAscan‐SE version 2.0.2^37^ and RNAmmer version 1.2^38^, respectively. Detection of the CRISPR‐Cas system in the genomes of *Thermus* was done using the CRISPRCasFinder tool (https://crisprcas.i2bc.paris-saclay.fr)^39^. The ViroBLAST (http://indra.mullins.microbiol.washington.edu/viroblast/viroblast.php)^40^ was used to identify the targets of the spacers, while the nonsimilar spacers were analyzed using BLAST against the NT database. The ISsaga software (http://issaga.biotoul.fr/issaga_index.php)^41^ was used to determine the IS elements, and the IslandPath‐DIMOB^42^ was used to predict GI, to provide the evidence of horizontal transfer regions.

### Phylogenetic analysis

A reference genomic dataset of the genus *Thermus* was established by downloading type strain genomes from NCBI (Table S3). The genomes with estimated completeness >95% and contamination <5% were kept for phylogenetic analysis. The phylogeny of *Thermus* was generated as mentioned in our previous study^43^, ^44^. Briefly, GTDB‐Tk software^12^ was used to generate the multiple sequence alignments (MSAs) with 120 bacterial marker genes, and IQ‐Tree^45^ was used for calculating a maximum‐likelihood phylogeny for MSAs with parameters (‐alrt 1000 ‐bb 1000 ‐nt AUTO). The best‐fit model (LG + F + R10) determined by ModelFinder^46^ was chosen according to Akaike Information Criterion (AIC), Corrected Akaike Information Criterion (Corrected AIC), and Bayesian Information Criterion (BIC).

For phylogeny based on genes of the denitrification pathway, datasets were derived from the NCBI reference genomes. The genomes were annotated as the methods above. The NarG protein sequences with length >1000 aa and <1300 aa were retained for this study. Reference NirK protein datasets with sequences longer than 400 aa were kept for further analysis. Similarly, full‐length NirS (>400 aa), NorB with the length of 450–850 aa, and NosZ with the average length of 662 aa were kept. Finally, representative sequences for each dataset (Tables S7–S11) were aligned using MUSCLE^47^ with 100 iterations. Phylogenetic trees were constructed using IQ‐Tree^45^ with the parameters mentioned above. All trees were visualized and annotated using iTOL^48^.

### Gene content comparison

Pan‐ and core‐genome analysis was performed using the Roary pipeline^49^ at 40% minimum sequence identity. The ANI among the genomes of genus *Thermus* was calculated by using the pyANI^50^ with BLAST method. Orthologous proteins were identified based on reciprocal BLAST best hits of predicted amino acid sequences (*E*‐value < 1e−5), and AAI of each pair of genomes was calculated as the mean identity of all orthologous proteins. All plots were generated with R^51^ with the ggplot2 package^52^. A Bayesian tree based on MSAs was constructed with the parameters (ngen = 3000000 Nruns = 2 Nchains = 4 diagnfreq = 1000 relburnin = yes burninfrac = 0.25 samplefreq = 100 printfreq = 100)^43^ by using MrBayes software^19^. The evolutionary history of the phylum *Deinococcota* was inferred by COUNT^20^ as described previously^43^, ^53^.

## AUTHOR CONTRIBUTIONS

Wen‐Jun Li and Brian P. Hedlund jointly conceived the study. Jian‐Yu Jiao conceptualized the research goals under the supervision of Wen‐Jun Li and Brian P. Hedlund, Jian‐Yu Jiao, Zheng‐Han Lian, Meng‐Meng Li, En‐Min Zhou, Lan Liu, and Hong Ming performed the bioinformatics analyses. Jian‐Yu Jiao and Zheng‐Han Lian prepared the figures and tables. Jian‐Yu Jiao, Zheng‐Han Lian, Meng‐Meng Li, Nimaichand Salam, Brian P. Hedlund, and Wen‐Jun Li wrote the manuscript. All authors revised and approved the final manuscript.

## ETHICS STATEMENT

This article does not contain any studies with human participants or animals performed by any of the authors. 

## CONFLICT OF INTERESTS

The authors declare that they have no conflict of interests.

## Supporting information

Supporting information.

Supporting information.

Supporting information.

Supporting information.

Supporting information.

Supporting information.

Supporting information.

Supporting information.

Supporting information.

Supporting information.

Supporting information.

Supporting information.

Supporting information.

Supporting information.

Supporting information.

## Data Availability

All the genomes described in this article have been deposited in NCBI, and the accession numbers were provided in Table S3. The Thermus information can also be found in eLMSG (an eLibrary of Microbial Systematics and Genomics, https://www.biosino.org/elmsg/index) under accession numbers MSG069325, MSG066408, MSG019978, MSG066894, MSG067219, MSG066285, MSG068958, MSG065983, MSG068209, MSG065453, MSG067283, MSG007907. The R codes have been deposited in Github (https://github.com/lianzhh-pub/Rcode-mLife2021).
