# Supplementary material for: Comparative genomic analysis of Thermus provides insights into the evolutionary history of an incomplete denitrification pathway
Source: mLife. 2022 Apr 29;1(2):198–209. doi: 10.1002/mlf2.12009 (PMC10989939; doi:10.1002/mlf2.12009)
Supplement: Supplementary file 1 — Supporting information. [file MLF2-1-198-s007.pdf]

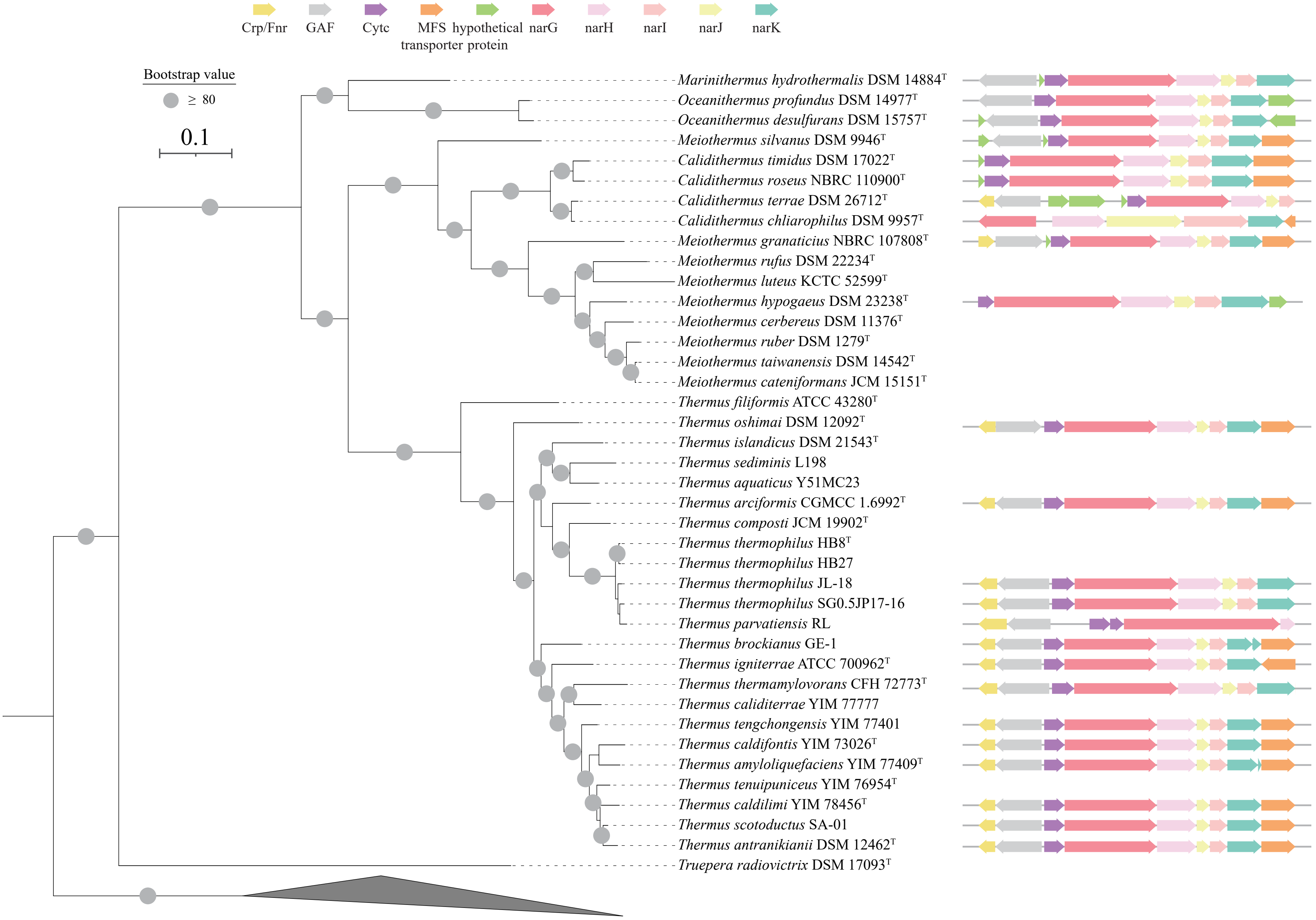

Crp/Fnr
GAF
Cytc
MFS transporter
hypothetical protein
narG
narH
narI
narJ
narK
Bootstrap value
≥ 80
0.1
Marinithermus hydrothermalis DSM 14884T
Oceanithermus profundus DSM 14977T
Oceanithermus desulfurans DSM 15757T
Meiothermus silvanus DSM 9946T
Calidithermus timidus DSM 17022T
Calidithermus roseus NBRC 110900T
Calidithermus terrae DSM 26712T
Calidithermus chliarophilus DSM 9957T
Meiothermus granaticius NBRC 107808T
Meiothermus rufus DSM 22234T
Meiothermus luteus KCTC 52599T
Meiothermus hypogaeus DSM 23238T
Meiothermus cerbereus DSM 11376T
Meiothermus ruber DSM 1279T
Meiothermus taiwanensis DSM 14542T
Meiothermus cateniformans JCM 15151T
Thermus filiformis ATCC 43280T
Thermus oshimai DSM 12092T
Thermus islandicus DSM 21543T
Thermus sediminis L198
Thermus aquaticus Y51MC23
Thermus arciformis CGMCC 1.6992T
Thermus composti JCM 19902T
Thermus thermophilus HB8T
Thermus thermophilus HB27
Thermus thermophilus JL-18
Thermus thermophilus SG0.5JP17-16
Thermus parvatiensis RL
Thermus brockianus GE-1
Thermus igniterrae ATCC 700962T
Thermus thermamylovorans CFH 72773T
Thermus caliditerrae YIM 77777
Thermus tengchongensis YIM 77401
Thermus caldifontis YIM 73026T
Thermus amyloliquefaciens YIM 77409T
Thermus tenuipuniceus YIM 76954T
Thermus caldilimi YIM 78456T
Thermus scotoductus SA-01
Thermus antranikianii DSM 12462T
Truepera radiovictrix DSM 17093T
