# Supplementary figures and images for: Comparative genomic analysis of Thermus provides insights into the evolutionary history of an incomplete denitrification pathway
Source: mLife. 2022 Apr 29;1(2):198–209. doi: 10.1002/mlf2.12009 (PMC10989939; doi:10.1002/mlf2.12009)

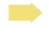 Crp/Fnr   
 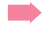 CytC   
 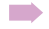 hypothetical protein   
 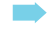 MarR   
 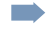 nirK   
 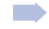 nirS   
 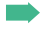 norB   
 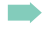 norC   
 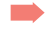 nrdR   
 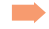 Rrf2   
 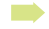 TIGR04053   
 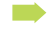 YbaN

Bootstrap value  
 ● ≥ 80  
 0.1

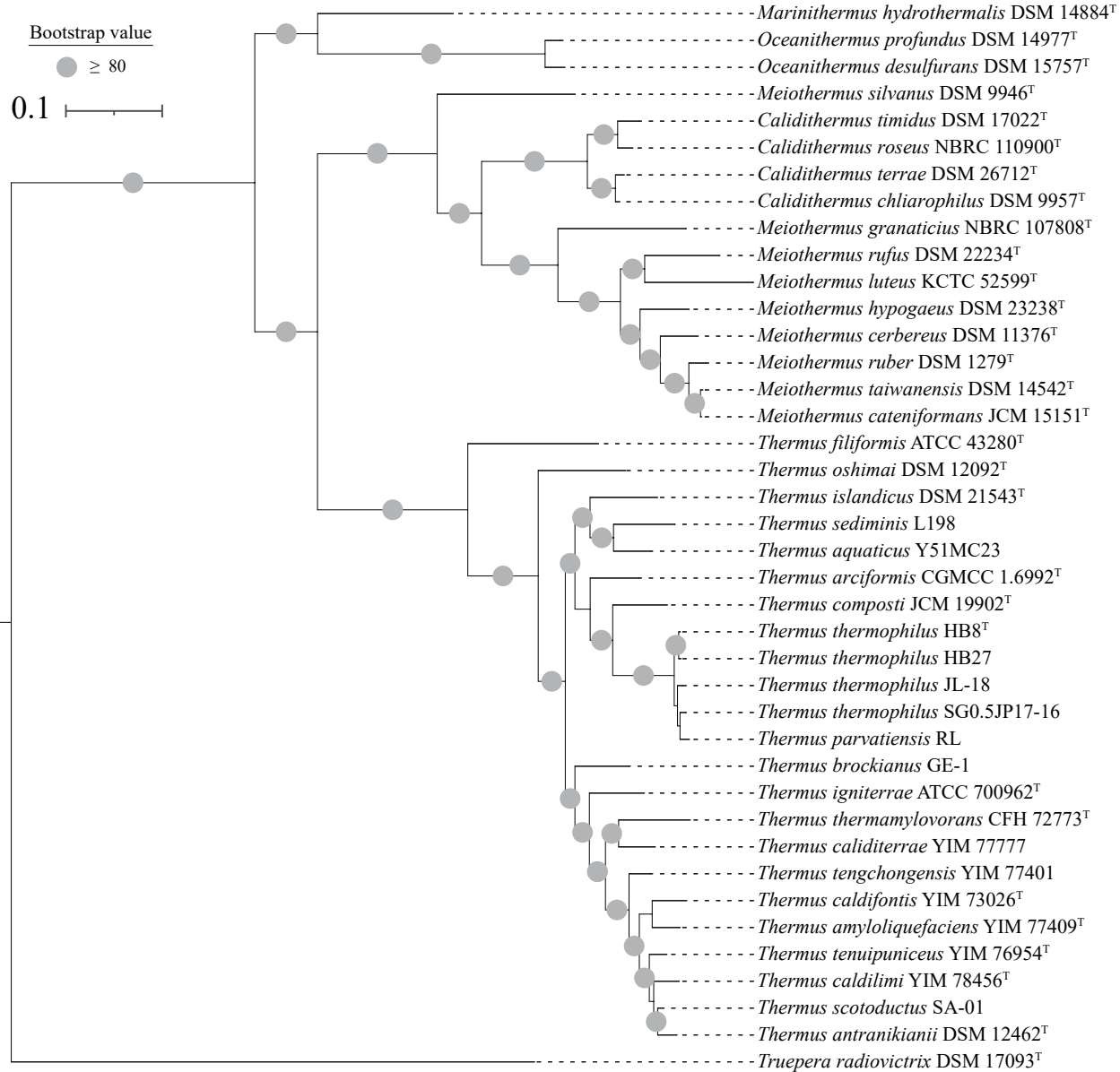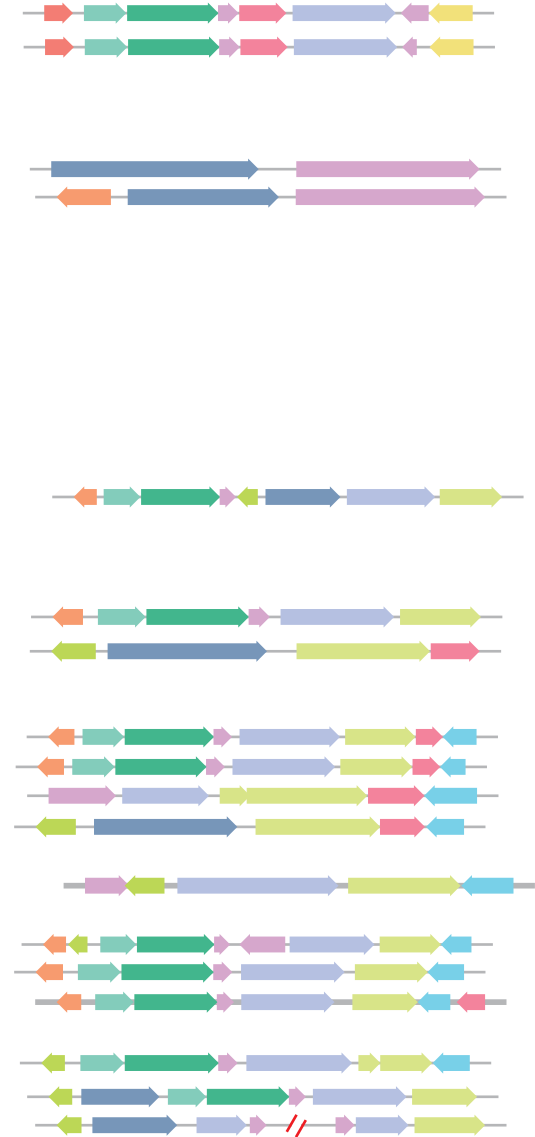

Supplement: Supplementary file 2 — Supporting information. [file MLF2-1-198-s003.pdf]

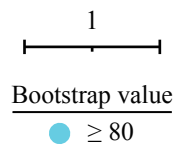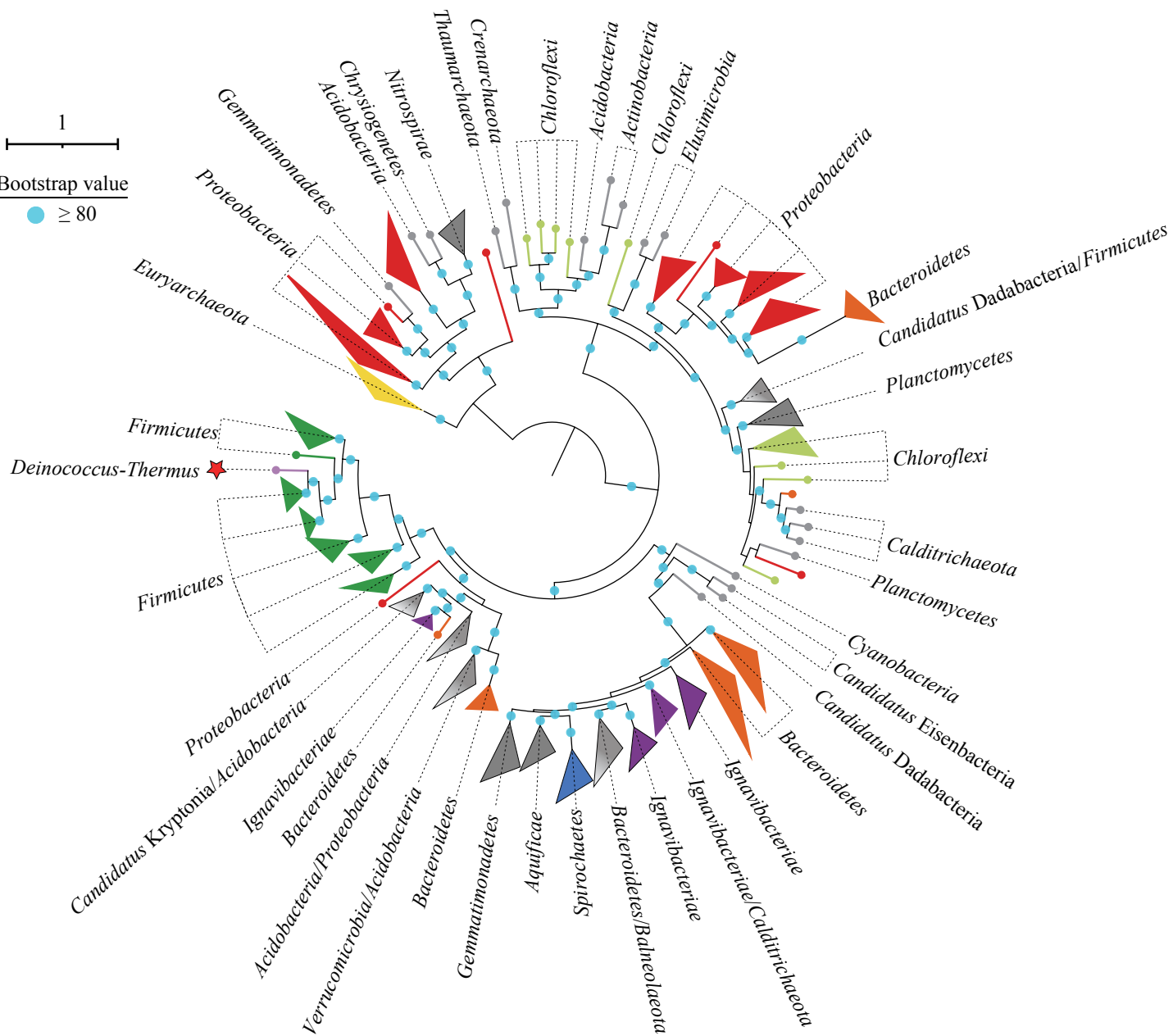

Supplement: Supplementary file 3 — Supporting information. [file MLF2-1-198-s009.pdf]
