# Supplementary material for: Comparative genomic analysis of Thermus provides insights into the evolutionary history of an incomplete denitrification pathway
Source: mLife. 2022 Apr 29;1(2):198–209. doi: 10.1002/mlf2.12009 (PMC10989939; doi:10.1002/mlf2.12009)
Supplement: Supplementary file 15 — Supporting information. [file MLF2-1-198-s008.docx]

**Supplementary Figures and Tables**

**Figure S1. Gene clusters related to *narGHI*.**

**Figure S2. Gene clusters related to *nirK*, *nirS,* and *norBC*.**

**Figure S3. Maximum-likelihood phylogeny of NosZ.** The red star represents the NosZ of *Deinococcus ficus* CC-FR2-10^T^. The NosZ protein sequences listed in Table S11 were aligned using MUSCLE (45) with 100 iterations. The NosZ tree was constructed using IQ-Tree (43) with the parameters (-alrt 1000 -bb 1000 -nt AUTO). The best-fit model (LG+F+R10) was determined by ModelFinder (44).

**Table S1. Genomic information of *Deinococcota* strains.**

**Table S2. Protein sequences of NarG for Figure 4.**

**Table S3. Protein sequences of NirK for Figure 5.**

**Table S4. Protein sequences of NirS for Figure 6.**

**Table S5. Protein sequences of NorB for Figure 7.**

**Table S6. Protein sequences of NosZ for Figure S3.**

**Table S7. Average nucleotide identity (ANI) values among the *Thermus* species.**

**Table S8. Average amino acid identity (AAI) values among the *Thermus* species.**

**Table S9. Genomic features of insertion sequence (IS) elements of *Thermus*.**

**Table S10. Genomic features of genomic islands (GI) of *Thermus*.**

**Table S11. The number of genes in *Thermus* genomes annotated to different COG functional categories.**
